# Ambient temperature, rainfall, and adverse maternal and child health outcomes in Nigeria: evidence from a national cross-sectional study

**DOI:** 10.3389/fpubh.2026.1841642

**Published:** 2026-06-29

**Authors:** Asri Maharani, Tung Le, Jahanara Miah, Zahra Mulla, Amy Blakemore, Deborah Aluh, John Jemisenia, Ngozi Idemili-Aronu, Echezona Edozie Ezeanolue, Martie van Tongeren, Rathi Ravindrarajah

**Affiliations:** 1 Division of Nursing, Midwifery and Social Work, University of Manchester, Manchester, United Kingdom; 2 Department of General Planning, Hanoi Medical University Hospital, Hanoi Medical University, Hanoi, Vietnam; 3 Division of Neuroscience, University of Manchester, Manchester, United Kingdom; 4 Division of Medical Education, University of Manchester, Manchester, United Kingdom; 5 School of Health, Sciences, and Society, University of Greater Manchester, Bolton, United Kingdom; 6 IVAN Research Institute, Enugu, Nigeria; 7 University of Nigeria, Nsukka, Nigeria; 8 Division of Population Health, Health Services Research and Primary Care, University of Manchester, Manchester, United Kingdom

**Keywords:** ambient temperature, child undernutrition, climate change, environmental epidemiology, maternal health, Nigeria

## Abstract

**Background:**

Climate change increasingly threatens public health in West Africa, with pregnant women and young children particularly vulnerable. Despite Nigeria’s high exposure to climate risks, epidemiological evidence linking temperature and rainfall to maternal and child health remains limited. This study addresses this gap using nationally representative data.

**Methods:**

We analysed data from the 2024 Nigeria Demographic and Health Survey, including 27,783 mother–child pairs. Climate exposures, i.e., daytime land surface temperature (°C) and annual rainfall (mm), were derived from the 2020 Nigeria Geospatial Covariates dataset and linked to 2024 DHS cluster geolocations. Child health outcomes included stunting, wasting, underweight, and fever. Maternal outcomes included anaemia, postpartum distress, and a composite healthcare access index. Multilevel mixed-effects regression models with survey weights were applied.

**Results:**

Higher temperatures were associated with increased odds of stunting (aOR 1.12, 95% CI 1.04–1.22), wasting (aOR 1.15, 95% CI 1.02–1.29), underweight (aOR 1.12, 95% CI 1.04–1.21), and fever (aOR 1.08, 95% CI 1.01–1.16) in children. Among mothers, higher temperatures were linked to greater postpartum distress (*β* = 0.03, *p* < 0.05) and reduced healthcare access (*β* = −0.07, *p* < 0.01), but not anaemia. A negative interaction between temperature and rainfall suggested attenuation of heat effects on underweight and healthcare access in wetter areas. Poverty and low maternal education amplified risks.

**Conclusion:**

Higher temperatures are associated with poorer maternal and child health outcomes in Nigeria, particularly among socioeconomically disadvantaged groups. Integrating climate adaptation into maternal and child health programmes is essential, especially in high-risk regions.

## Introduction

Climate change is widely recognised as one of the most consequential public health threats of the 21st century. The World Health Organisation (WHO) estimates that climate change will lead to about 250,000 additional deaths between 2030 and 2050 from malnutrition, malaria, diarrhoea, and heat stress, disproportionately affecting countries with weak health systems (1). Despite contributing the least to global greenhouse gas emissions, low- and middle-income countries (LMICs) face the greatest health impacts. In the Africa region, climate-related effects account for 34% of disability-adjusted life years (DALYs), nearly three times greater than the region’s 11% share of the global population (2).

Although LMICs, including African countries such as Nigeria, contribute comparatively little to global emissions, they experience the most severe impacts, including altered disease patterns, threats to food and water security, and increasing frequency of extreme weather events (3). Africa has experienced an over 1 °C rise in temperature since 1901, driving more frequent and intense heatwaves, and Nigeria faces a constellation of overlapping climate hazards, including extreme heat in the arid north, erratic and intensifying rainfall, desertification, and recurrent flooding. Projections for Nigeria suggest that climate change will substantially increase the burden of diarrhoeal mortality, cardiovascular disease, diabetes, and mental health disorders by 2030 (4).

Pregnant women and young children are among the populations most vulnerable to these climate-related health impacts. The African region alone contributes approximately 70% of global maternal deaths, and climate change further increases vulnerability to adverse maternal health outcomes (5) through both direct physiological mechanisms and indirect pathways. Flooding has been associated with maternal anaemia, preeclampsia, and eclampsia through several indirect pathways. With respect to anaemia, flooding contaminates drinking water sources and increases exposure to waterborne pathogens, triggering systemic inflammation that impairs iron metabolism, whilst simultaneously compromising dietary diversity and reducing micronutrient availability, including retinol, through disruption of food systems (6). Furthermore, flooding creates extensive stagnant water bodies that serve as breeding sites for Anopheles mosquitoes, amplifying malaria transmission in flood-affected communities (7). In Nigeria, which bears one of the world’s highest burdens of malaria-attributable maternal anaemia, Plasmodium falciparum infection during pregnancy drives haemolysis and suppresses erythropoiesis, substantially elevating the risk of anaemia in pregnant women (8, 9). With respect to hypertensive disorders of pregnancy, the acute psychosocial stress associated with flood-related displacement, property loss, and livelihood disruption activates the hypothalamic–pituitary–adrenal axis, elevating circulating cortisol levels; sustained cortisol elevation is associated with endothelial dysfunction and hypertension, both central features of preeclampsia and eclampsia (10). Additionally, flooding disrupts road networks and healthcare infrastructure, reducing pregnant women’s access to antenatal monitoring at precisely the moments when early identification and management of hypertensive disorders are most critical (10, 11). Disruption to food and water systems drives malnutrition and dehydration in pregnant women, increasing the risk of adverse birth outcomes, including preterm delivery and developmental impairment in newborns. Flooding contaminates water sources, elevating exposure to waterborne pathogens such as cholera and typhoid infections that carry heightened risks for both mother and foetus. Flood waters also introduce environmental toxins and pollutants that may compromise foetal development and maternal health.

For child health, the evidence is increasingly consistent. High temperatures and low precipitation are associated with reductions in child weight and increased risk of wasting across the African continent (12), operating through disruptions to food systems, agricultural productivity, and water safety. Projections suggest that by 2,100, child wasting could increase by 37% in Africa due to rising temperatures (13), threatening to reverse decades of hard-won progress in reducing child undernutrition.

Those detrimental effects of climate change extend beyond physical health. Exposure to high temperatures has been associated with a statistically significant increase in the risk of postpartum depression, with the greatest risk among women simultaneously exposed to high levels of air pollution and limited access to cooling (14), conditions far more prevalent in LMICs than in high-income countries. Extreme weather events have also been shown to increase the risk of postpartum depression and post-traumatic stress disorder, as well as preterm birth and low birthweight (15). Beyond the physical environment, extreme weather events destabilise healthcare infrastructure, disrupting access to prenatal care at precisely the moments it is most needed. The cumulative psychological burden of climate-related displacement and loss compounds these risks further, with evidence linking maternal stress to poor birth outcomes (16, 17).

Despite this high burden, existing studies from Nigeria have tended to examine child malnutrition or maternal anaemia in isolation, rarely considering climate as an underlying driver (18, 19). Climate-health research in Nigeria has largely focused on agricultural and economic impacts, with the few health-focused studies limited to small, localised samples that cannot speak to national patterns (20–22). To date, limited studies have examined how temperature and rainfall jointly influence child nutritional status, infectious morbidity, maternal postpartum distress, anaemia, and healthcare access within a single nationally representative framework, nor have any Nigerian studies assessed how socioeconomic factors amplify or attenuate these climate-related health risks. This study addresses these gaps using data from the 2024 Nigeria Demographic and Health Survey linked to geospatial climate covariates. Using multilevel mixed-effects regression models, we examine associations between daytime land surface temperature and annual rainfall and a comprehensive set of maternal and child health outcomes and identify household and community-level factors that modify these relationships.

## Materials and methods

### Study design and participants

Data from the 2024 Nigeria Demographic and Health Survey (DHS), a nationally representative cross-sectional survey, were used for this study. The 2024 Nigeria DHS was conducted by the National Population Commission (NPC) of Nigeria and ICF International, and the dataset is publicly available following a formal application to the DHS Programme. This dataset can be downloaded from: https://dhsprogram.com/data/available-datasets.cfm. The DHS employed a complex, stratified two-stage cluster sampling design. In the first stage, primary sampling units, or geographic clusters, were selected from urban and rural strata across Nigeria using a probability proportional to size method. In the second stage, households were selected within each designated cluster via systematic random sampling. This sampling architecture inherently generates a hierarchical, nested data structure, wherein individual mothers and children (Level 1) are nested within households, which are further nested within geographic clusters (Level 2). Although households constitute an intermediate organisational unit within the DHS sampling frame, household-level random effects were not modelled as a separate third level, as the dominant source of hierarchical variation in DHS-based analyses of this type operates at the cluster level, and two-level specifications are standard in the DHS-linked multilevel literature (23, 24). This multi-stage hierarchical nesting directly justifies the application of multilevel mixed-effects models to account for intra-cluster correlation and ensure precise variance estimation. The study population comprised mother–child pairs, defined as all living children aged 0–59 months and their mothers residing in the same household. Consistent with the DHS survey methodology, analytical samples for designated outcomes were limited to households chosen for anthropometric assessments and selected maternal health modules, thereby maintaining data integrity.

### Climate exposure

The environmental exposure was assessed by linking DHS cluster geolocations to the 2020 Nigeria Geospatial Covariates dataset. The 2020 Nigeria Geospatial Covariates dataset was selected as it represents the most recent validated geospatial dataset produced by the DHS Programme that is spatially compatible with the Nigerian DHS cluster framework. No equivalent validated dataset is available for subsequent years. The approach of utilising non-contemporaneous baseline climate data or multi-year spatial environmental proxies to study chronic and structural health outcomes in cross-sectional surveys is well-established in the DHS-linked literature (25–27). For instance, Randell et al. linked DHS child anthropometric outcomes in Ethiopia to weather data spanning multiple years before the survey interview (25), explicitly incorporating lagged in-utero and early-life exposure windows of up to 5 years before outcome measurement. Similarly, Grace et al. matched DHS birth weight records from 19 African countries to satellite-derived climate data from periods predating the outcome assessments by up to several years (26), demonstrating that temporally offset climate-health linkages are methodologically established in this literature. These precedents directly support our use of the 2020 geospatial covariates as a valid baseline exposure reference for health outcomes measured in 2024. Climate variables derived from this dataset primarily capture the structural geographic distribution of temperature and rainfall across Nigeria, a pattern characterised by stable north–south gradients in thermal exposure and precipitation that are unlikely to have changed substantially in direction or relative magnitude between 2020 and 2024. Accordingly, these variables are interpreted as indicators of chronic regional climate exposure rather than year-specific conditions.

Daytime land surface temperature (LST) was measured in degrees Celsius (°C) to assess heat stress. It is acknowledged that daytime LST, derived from satellite imagery, can differ substantially from ambient air temperature experienced at ground level, with discrepancies of up to 10–15 °C reported in some settings (28, 29). LST is particularly poorly correlated with indoor temperatures, which are likely most relevant to outcomes such as postpartum distress and infant health. Its use in this study reflects the constraints of data availability within the DHS geospatial framework. No validated cluster-level ambient air temperature data were available for Nigeria for the study period. Total annual rainfall was measured in millimetres (mm) to reflect moisture availability. Climate variables were treated as continuous measures.

### Outcome measures

Child health was evaluated using three anthropometric indicators based on WHO Child Growth Standards, including stunting (chronic malnutrition), defined as a Height-for-Age Z-score (HAZ) < −2 SD; wasting (acute malnutrition), defined as a Weight-for-Height Z-score (WHZ) < −2 SD; and underweight (composite nutritional status), defined as a Weight-for-Age Z-score (WAZ) < −2 SD (30). Additionally, fever was assessed as a binary indicator of whether the child had a fever in the 2 weeks preceding the survey.

Postpartum distress was assessed using a four-item mental health module included in the 2024 Nigeria DHS women’s questionnaire. The module comprised four self-reported items asking respondents whether, in the past 2 weeks, they had experienced: feeling anxious or worried; feeling depressed or hopeless; loss of interest or pleasure in activities (anhedonia); and thoughts of self-harm or suicide. Responses were summed and standardised into a z-score, with higher values indicating greater distress. This module is not a validated instrument for postpartum depression and does not correspond to established clinical screening tools such as the Edinburgh Postnatal Depression Scale (EPDS) or the Patient Health Questionnaire (PHQ-9). Consequently, due to the absence of validated postpartum mental health instruments in the DHS, this measure was utilised as an indicator of general psychological distress rather than as an estimate of postpartum depression prevalence. Maternal anaemia was defined using the standard thresholds applied by the DHS Programme and consistent with WHO public health surveillance guidelines: haemoglobin below 12.0 g/dL for non-pregnant women and below 11.0 g/dL for pregnant women (31). These thresholds reflect the DHS Programme’s operational classification approach and do not distinguish women in the immediate postpartum period from other non-pregnant women. The DHS Programme does not apply a separate haemoglobin threshold for women in the immediate postpartum period (first 6 weeks after delivery), and postpartum women are classified according to their pregnancy status at the time of haemoglobin measurement. Women identified as non-pregnant at the time of the survey were therefore assigned the 12.0 g/dL threshold, regardless of time since delivery. It is acknowledged that WHO public health surveillance guidelines recommend applying the lower threshold of < 11.0 g/dL to women within the first 6 weeks postpartum, recognising their physiological similarity to pregnant women (31, 32), and that the DHS classification therefore diverges from this recommendation for recently delivered women. Given that the DHS biomarker data do not include a variable identifying women within this specific postpartum window, applying a differential threshold was not feasible within this dataset. This classification approach is consistent with standard practise in DHS-based anaemia research (33, 34) and is noted as a limitation of the data. Finally, healthcare access was operationalised as a composite index derived via Principal Component Analysis (PCA) of three binary indicators: antenatal care attendance (4 + visits), tetanus toxoid vaccination, and institutional delivery (35).

### Covariates

This study included several covariates representing key demographic and socioeconomic characteristics. Demographic controls included child age (in months) to account for growth trajectories and the timing of postpartum recovery, child biological sex, and maternal parity (total number of children ever born). Socioeconomic status was approximated using the DHS Wealth Index, divided into five national quintiles (Poorest to Richest), and maternal education was categorised into four levels (No education, Primary, Secondary, Higher). The place of residence was classified as urban or rural. Maternal age (in years) was included as a control variable in the maternal health models.

### Statistical analysis

Descriptive statistics were computed for all study variables using survey weights to account for the complex sampling design. Continuous variables, including child age, maternal age, maternal parity, daytime land surface temperature, annual rainfall, postpartum distress z-score, and healthcare access index, are presented as weighted means with standard deviations. Categorical variables, including child sex, place of residence, maternal education, household wealth quintile, and all binary health outcomes, are presented as weighted frequencies and percentages. Bivariate associations between exposures and outcomes were examined separately for child and maternal outcomes. For associations between categorical predictors and binary outcomes, chi-squared tests were used, which adjust the standard Pearson chi-squared statistic for the complex survey design.

We employed multilevel mixed-effects models to account for the hierarchical structure of the DHS data, in which mother–child pairs (level 1) were nested within enumeration clusters (level 2). This approach is appropriate because observations within the same cluster are unlikely to be independent, violating the assumptions of standard regression models, and failing to account for this clustering would underestimate standard errors and inflate the precision of estimates. A random intercept was included at the cluster level to capture unobserved community-level heterogeneity, for example, shared environmental conditions, healthcare infrastructure, and cultural practises that may influence health outcomes independently of the measured covariates.

Considering individuals *i* nested within clusters *j*, the general model takes the form:

For binary outcomes (stunting, wasting, underweight, fever, anaemia):

$$\text{logit}\;(P(Y_{\mathrm{ij}}=1))=\beta_{00}+\Sigma \gamma_{\mathrm{oj}}W_{j}+\Sigma \beta_{\mathrm{ki}}X_{\mathrm{ij}}+u_{\mathrm{oj}}+{\varepsilon_{\mathrm{ij}}}^{*}$$

where:

- *Y*_ij_ is the binary health outcome for individual *i* in cluster *j*
- *W*_j_ is the set of cluster-level climate exposures (daytime temperature and annual rainfall)
- *X*_ij_ is the set of individual- and household-level covariates (child age, sex, maternal education, parity, household wealth, place of residence)
- *u*_oj_ is the random intercept varying over clusters, with mean zero and variance σ^2^ᵤ, capturing unobserved cluster-level variation
- *ε*_ij_ is the residual error term, assumed normally distributed with mean zero

Results for binary outcomes are reported as Adjusted Odds Ratios (aOR) with 95% Confidence Intervals (CI).

For continuous outcomes (postpartum distress z-score, healthcare access index):

$$Y_{\mathrm{ij}}=\beta_{00}+\Sigma Y_{\mathrm{oj}}W_{j}+\Sigma \beta_{\mathrm{ki}}X_{\mathrm{ij}}+U_{\mathrm{oj}}+\varepsilon_{\mathrm{ij}}$$

where all terms are as above, and *Y*_ij_ is the continuous outcome. Results are reported as regression coefficients (β) with 95% CIs.

All models included a temperature × rainfall interaction term to assess whether the effect of heat on health outcomes was modified by moisture availability. Survey sampling weights were applied to all analyses to account for the complex sampling design and ensure national representativeness. The intraclass correlation coefficient (ICC) was calculated for each model to quantify the proportion of total outcome variance attributable to cluster-level factors. Data analysis was conducted using Stata 19.

## Results

**Figure 1:**
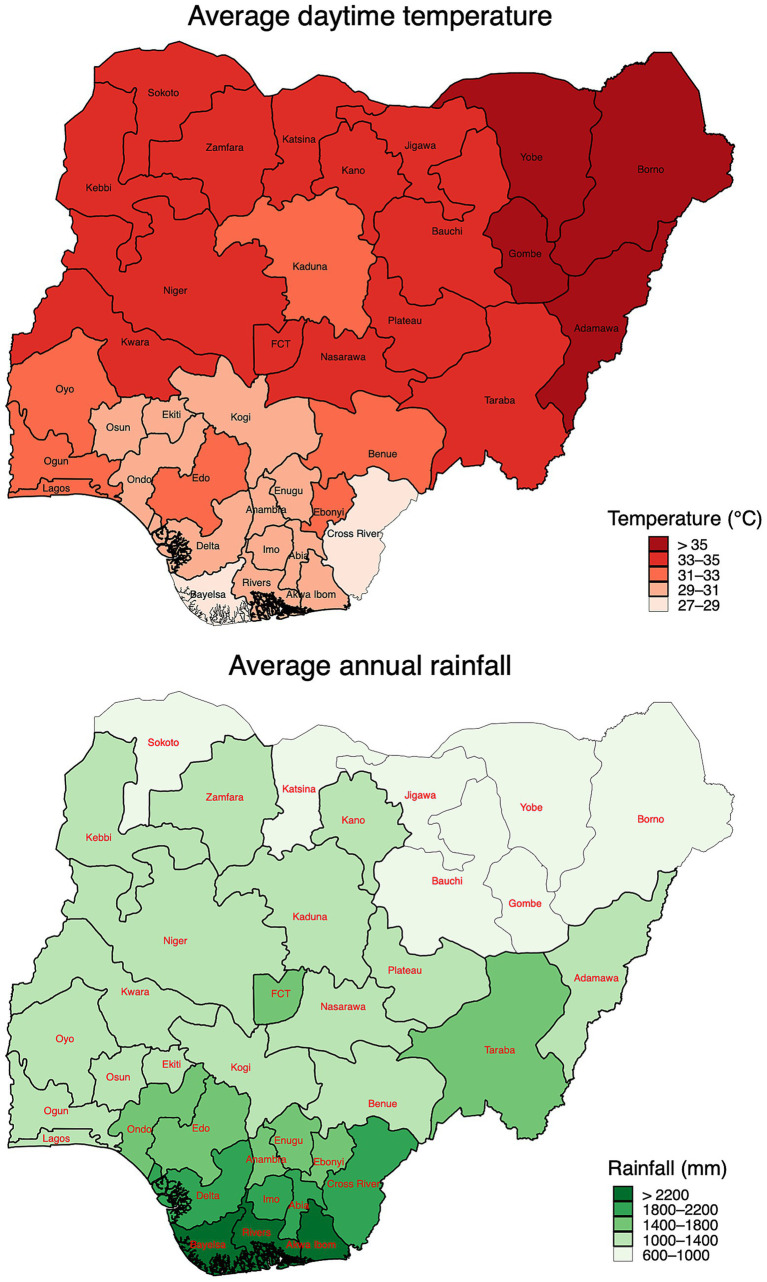
Geographical distribution of average daytime temperature (°C) and annual rainfall (mm) across 36 states and the Federal Capital Territory of Nigeria.

Figure 1 describes the geographical distribution of climate exposure across 36 states and the Federal Capital Territory (FCT) of Nigeria. The maps highlight the variations in environmental conditions, with a distinct inverse relationship between temperature and rainfall. Regarding thermal exposure, the highest average daytime temperatures are found across the Northern zones, specifically in the states of Borno, Yobe, and Sokoto, where values exceed 35 °C. In contrast, the lowest temperatures are concentrated in the central highlands, distinctively within Plateau State. The distribution of rainfall shows an opposing trend. The highest precipitation levels occur along the coastal states of the South South and South West zones, most notably in Bayelsa, Delta, Rivers, and Akwa Ibom, where annual rainfall surpasses 2,200 mm. This precipitation decreases progressively towards the arid northern border.

Table 1 presents the weighted characteristics of the 27,783 children and mothers included in the study. The study population was predominantly rural (61.6%), with nearly half of households falling into the poorest or poorer wealth quintiles (47.1%). Maternal education levels were generally low, with 47.0% of mothers having no education, whilst only 9.9% had attained higher education. The average maternal parity was 4.1 children (SD 2.5). Regarding climate exposures, the mean daytime land surface temperature was 33.3 °C (SD 2.3), and the mean annual rainfall was 1204.9 mm (SD 403.1). In terms of health outcomes, stunting was highly prevalent, with 39.1% of children classified as stunted. Additionally, 26.4 and 8.4% were underweight and wasted, respectively. Fever was reported for 15.9% of children. Among mothers, 47.5% were anaemic. The mean postpartum distress score was −0.02 (SD 0.98), and the mean healthcare access index was −0.06 (SD 1.39), confirming that these measures were standardised with a mean approximating zero.

**Table 1:** Sociodemographic, climatic, and health characteristics of the study population (*N* = 27,783).

Variable	*n* (%) or mean (SD)	Missing data n (%)
Climate exposures		
Daytime temperature (°C)	33.3 (2.3)	0 (0.0)
Annual rainfall (mm)	1204.9 (403.1)	6 (<0.1)
Child characteristics		
Child age (months)	29.96 (17.84)	0 (0.0)
Child sex		0 (0.0)
Male	14,570 (50.72)	
Female	14,158 (49.28)	
Maternal and household characteristics		
Mother’s age in years	29.99 (6.88)	0 (0.0)
Mother’s education		0 (0.0)
No education	13,489 (46.95)	
Primary	3,413 (11.88)	
Secondary	8,974 (31.24)	
Higher	2,852 (9.93)	
Maternal parity (total children)	4.13 (2.50)	0 (0.0)
Household wealth quintile		0 (0.0)
Poorest	7,013 (24.41)	
Poorer	6,522 (22.70)	
Middle	5,802 (20.19)	
Richer	5,042 (17.55)	
Richest	4,349 (15.14)	
Place of residence		0 (0.0)
Urban	11,039 (38.43)	
Rural	17,689 (61.57)	
Child health outcomes		
Stunting		18,373 (66.1)
Not stunted	5,685 (60.86)	
Stunted	3,656 (39.14)	
Wasting		18,319 (65.9)
Not wasted	8,609 (91.56)	
Wasted	793 (8.44)	
Underweight		18,270 (65.8)
Not underweight	6,953 (73.59)	
Underweight	2,496 (26.41)	
Fever		2,307 (8.3)
No	22,005 (84.09)	
Yes	4,164 (15.91)	
Maternal health outcomes		
Anaemia (any)		16,730 (60.2)
No	2,994 (52.63)	
Yes	2,695 (47.37)	
Postpartum distress score (z-score)	−0.02 (0.98)	13,743 (49.5)
Healthcare access index (PCA)	−0.06 (1.39)	14,263 (51.3)

Bivariate analyses (Supplementary Tables S1, S2) revealed significant associations between climate exposures and health outcomes. Children classified as stunted or underweight resided in areas with significantly higher mean daytime temperatures and lower annual rainfall compared to their healthy counterparts (*p* < 0.001). Additionally, low household wealth and lack of maternal education were strongly linked to higher rates of child malnutrition and maternal anaemia (*p* < 0.001). Regarding maternal-specific outcomes, climate variables showed no statistically significant bivariate association with anaemia or postpartum distress scores. However, environmental conditions were strongly correlated with healthcare access; higher temperatures were associated with lower access scores (*β* = −0.14, *p* < 0.001), whilst higher rainfall was associated with better access scores (*β* = 0.001, *p* < 0.001). Urban residence was consistently associated with better healthcare access (*p* < 0.001).

Table 2 summarises the adjusted associations between climate, sociodemographic factors, and child health outcomes. Daytime temperature was positively associated with all four childhood health outcomes. Specifically, higher temperature was associated with higher odds of stunting (aOR 1.12, 95% CI 1.04–1.22), wasting (aOR 1.15, 95% CI 1.02–1.29), underweight (aOR 1.12, 95% CI 1.04–1.21), and fever (aOR 1.08, 95% CI 1.01–1.16). Regarding the interaction effects, a statistically significant negative interaction between temperature and rainfall was observed for underweight children (aOR 0.99, *p* < 0.01). Sociodemographic characteristics exhibited strong associations with child health metrics. Children in the wealthiest household quintile demonstrated lower odds of stunting (aOR 0.31, *p* < 0.001) and underweight (aOR 0.38, *p* < 0.001) relative to the poorest quintile. Higher maternal education was negatively associated with stunting, wasting, and underweight probabilities. Place of residence showed divergent patterns across nutritional indicators. Rural residence was associated with higher odds of stunting (aOR 1.29, 95% CI 1.09–1.53) but lower odds of wasting (aOR 0.77, 95% CI 0.62–0.96) compared to urban residence. Maternal parity did not show a statistically significant association with any of the child health outcomes.

**Table 2:** Associations between climate exposures and child health.

Variables	Stunting (aOR) (*n* = 9,409)	Wasting (aOR) (*n* = 9,463)	Underweight (aOR) (*n* = 9,512)	Fever (aOR) (*n* = 25,470)
Climate exposures				
Daytime temperature	1.12 [1.04, 1.22]**	1.15 [1.02, 1.29]*	1.12 [1.04, 1.21]**	1.08 [1.007, 1.16]*
Annual rainfall	1.001 [0.99, 1.003]	1.003 [1.0003, 1.005]*	1.002 [1.0005, 1.003]*	1.002 [1.0003, 1.003]*
Climate interaction (temperature × rainfall)	0.99 [0.99, 1.00]	0.99 [0.99, 1.00]	0.99 [0.9998, 0.9999]**	0.99 [0.99, 1.00]
Child characteristics				
Child age (months)	1.02 [1.02, 1.03]***	0.98 [0.97, 0.98]***	1.006 [1.002, 1.009]**	0.99 [0.991, 0.996]***
Sex				
Male	Ref.	Ref.	Ref.	Ref.
Female	0.74 [0.67, 0.83]***	0.90 [0.76, 1.07]	0.93 [0.83, 1.04]	0.96 [0.88, 1.05]
Sociodemographics				
Place of residence				
Urban	Ref.	Ref.	Ref.	Ref.
Rural	1.29 [1.09, 1.53]**	0.77 [0.62, 0.96]*	0.91 [0.77, 1.08]	1.09 [0.93, 1.27]
Maternal education				
No education	Ref.	Ref.	Ref.	Ref.
Primary	0.75 [0.60, 0.94]*	0.84 [0.59, 1.19]	0.79 [0.63, 0.99]*	1.05 [0.88, 1.25]
Secondary	0.73 [0.61, 0.89]**	0.85 [0.62, 1.17]	0.74 [0.61, 0.90]**	1.04 [0.89, 1.20]
Higher	0.40 [0.29, 0.55]***	0.51 [0.33, 0.78]**	0.38 [0.27, 0.53]***	0.99 [0.79, 1.23]
Maternal parity (total children)	1.007 [0.98, 1.03]	0.99 [0.95, 1.03]	1.006 [0.98, 1.03]	0.99 [0.97, 1.01]
Household wealth				
Poorest (quintile 1)	Ref.	Ref.	Ref.	Ref.
Poorer (quintile 2)	0.91 [0.76, 1.10]	0.87 [0.62, 1.20]	0.83 [0.69, 0.99]*	0.96 [0.83, 1.11]
Middle (quintile 3)	0.72 [0.59, 0.88]**	0.77 [0.54, 1.09]	0.65 [0.52, 0.80]***	1.02 [0.84, 1.23]
Richer (quintile 4)	0.60 [0.47, 0.76]***	1.06 [0.74, 1.53]	0.61 [0.48, 0.79]***	0.89 [0.71, 1.11]
Richest (quintile 5)	0.31 [0.22, 0.42]***	1.09 [0.72, 1.64]	0.38 [0.28, 0.53]***	0.86 [0.66, 1.12]
Random effects				
Area-level variance (SE)	0.51 (0.06)	0.54 (0.10)	0.39 (0.06)	0.63 (0.05)
Individual-level variance	Fixed (3.29)^†^	Fixed (3.29)^†^	Fixed (3.29)^†^	Fixed (3.29)^†^
ICC (%)	13.3	14.1	10.7	16.1

SE, Standard Errors; ICC, Intraclass Correlation Coefficient. ^†^For binary outcomes, the individual-level variance is fixed at π^2^/3 ≈ 3.29; *p < 0.05, **p < 0.01, ***p < 0.001.

The multilevel analysis revealed significant clustering effects. The Intraclass Correlation Coefficients (ICC) indicated that community-level factors accounted for a substantial proportion of the variance in child health outcomes, ranging from 10.7% for underweight to 16.1% for fever. This confirms that unobserved environmental or contextual factors at the cluster level play a critical role in shaping child nutritional status and disease susceptibility.

Table 3 presents the associations between climate exposures, sociodemographic factors, and maternal health outcomes. For anaemia, the effects of temperature and rainfall were not statistically significant. However, the interaction between temperature and rainfall was statistically significant (aOR 0.99, *p* < 0.01). Regarding mental health, elevated daytime temperature was positively associated with postpartum distress scores (*β* = 0.03, *p* < 0.05). Rainfall and the climate interaction were not significantly associated with postpartum distress. Conversely, both higher temperature (*β* = −0.07, *p* < 0.01) and annual rainfall (*β* = −0.001, *p* < 0.05) were associated with lower healthcare access scores, and a positive interaction between temperature and rainfall was also detected for this outcome (*p* < 0.05). Among sociodemographic variables, higher maternal education and household wealth were positively associated with healthcare access scores (*p* < 0.001). For postpartum distress, higher maternal education was associated with increased distress scores (*β* = 0.14 for higher education, *p* < 0.01). Older child age was associated with lower maternal distress scores (*β* = −0.003, *p* < 0.01). Rural residence was negatively associated with healthcare access (*β* = −0.29, *p* < 0.001) but showed no statistically significant association with anaemia or postpartum distress.

**Table 3:** Associations between climate exposures and maternal health.

Variables	Anaemia (aOR) (*n* = 7,200)	Postpartum distress (β) (*n* = 14,037)	Healthcare access (β) (*n* = 13,517)
Climate exposures			
Daytime temperature	1.07 [1.00, 1.15]	0.03 [0.003, 0.06]*	−0.07 [−0.11, −0.03]**
Annual rainfall	1.002 [1.0007, 1.004]**	0.0004 [−0.0002, 0.0009]	−0.001 [−0.002, −0.0002]*
Climate interaction (temperature × rainfall)	0.99 [0.9998, 0.9999]**	−0.00001 [−0.00003, 5.71e-06]	0.00004 [5.77e-06, 0.00007]*
Maternal and child characteristics			
Child age (months)	1.00 [0.99, 1.00]	−0.003 [−0.005, −0.001]**	−0.0003 [−0.002, 0.002]
Mother’s age (years)	0.99 [0.98, 1.01]	−0.002 [−0.007, 0.003]	0.012 [0.007, 0.02]***
Maternal parity (total children)	1.02 [0.99, 1.06]	−0.01 [−0.02, 0.004]	−0.04 [−0.05, −0.02]***
Sociodemographics			
Place of residence (Ref: Urban)			
Urban	Ref.	Ref.	Ref.
Rural	1.03 [0.88, 1.21]	0.02 [−0.04, 0.07]	−0.29 [−0.38, −0.21]***
Maternal education			
No education	Ref.	Ref.	Ref.
Primary	0.97 [0.78, 1.21]	0.06 [−0.02, 0.13]	0.35 [0.26, 0.43]***
Secondary	0.83 [0.68, 0.99]*	0.06 [−0.006, 0.12]	0.68 [0.60, 0.76]***
Higher	0.70 [0.54, 0.92]**	0.14 [0.04, 0.24]**	0.90 [0.80, 1.00]***
Household wealth			
Poorest (quintile 1)	Ref.	Ref.	Ref.
Poorer (quintile 2)	1.12 [0.92, 1.35]	−0.02 [−0.08, 0.04]	0.16 [0.08, 0.24]***
Middle (quintile 3)	0.93 [0.75, 1.16]	0.03 [−0.05, 0.10]	0.37 [0.28, 0.46]***
Richer (quintile 4)	1.08 [0.85, 1.37]	0.02 [−0.06, 0.11]	0.69 [0.58, 0.80]***
Richest (quintile 5)	0.88 [0.67, 1.16]	−0.01 [−0.11, 0.09]	0.91 [0.80, 1.02]***
Random effects			
Area-level variance (SE)	0.43 (0.06)	0.06 (0.01)	0.25 (0.01)
Individual-level variance	Fixed (3.29)^†^	0.88 (0.03)	0.94 (0.02)
ICC (%)	11.5	6.8	20.9

SE, Standard Errors; ICC, Intraclass Correlation Coefficient. ^†^For binary outcome, the individual-level variance is fixed at π^2^/3 ≈ 3.29; *p < 0.05, **p < 0.01, ***p < 0.001.

Regarding the random effects, the clustering of outcomes varied considerably. Healthcare access exhibited the strongest geographical influence (ICC = 20.9%), highlighting that a mother’s ability to access care is heavily dependent on her community of residence. Anaemia demonstrated moderate clustering (11.5%), whereas postpartum distress showed the lowest clustering effect (6.8%), suggesting that mental health is driven primarily by individual-level factors rather than community context.

## Discussion

This study examined the association between daytime land surface temperature, annual rainfall, and various maternal and child health outcomes in Nigeria using data from the 2024 Nigeria Demographic and Health Survey.

This study provides nationally representative evidence that elevated daytime temperatures exacerbate both maternal and child health vulnerabilities in Nigeria. Furthermore, our multilevel analysis confirms that community-level contexts independently drive variations in these outcomes, highlighting the critical role of the local environment. These results contribute to a growing but still limited body of evidence on the health dimensions of climate change in the African region (36, 37), and underscore the particular vulnerability of pregnant women, young children, and low-income households in Nigeria to the effects of rising temperatures (38).

The positive association between higher daytime temperatures and all four child health outcomes, i.e., stunting, wasting, underweight, and fever, is consistent with emerging evidence from comparable settings across the African continent (13, 39, 40). For example, using DHS data from 656,107 children across 52 surveys in 29 African countries linked to remotely sensed daytime land surface temperature, Tusting and colleagues found that temperatures above 35 °C were associated with significantly increased odds of wasting (OR 1.27, 95% CI 1.16–1.38) and underweight (OR 1.09, 95% CI 1.02–1.16), though stunting was inversely associated with temperature (OR 0.90, 95% CI 0.85–0.96) (13). There are several plausible mechanisms of the relationship between heat stress and child undernutrition. Elevated temperatures reduce agricultural productivity and food availability, directly compromising dietary intake and increasing the risk of chronic and acute malnutrition (13, 21). Heat exposure also increases metabolic demands on the body whilst simultaneously suppressing appetite, and may impair intestinal absorption through heat-induced gut permeability, sometimes referred to as “leaky gut,” which reduces the bioavailability of nutrients even when food intake is adequate (41). Furthermore, higher temperatures promote the proliferation of enteric pathogens in water and food supplies, increasing the burden of diarrhoeal disease, a major driver of wasting and underweight among young children (42). The association with fever is similarly consistent with known links between thermal stress, weakened immune function, and increased infectious disease burden (43), particularly in settings with limited access to safe water and sanitation. The high prevalence of chronic and acute malnutrition concentrated in the hotter northern regions of Nigeria aligns with these mechanistic pathways and highlights the compounding effect of chronic heat exposure on long-term growth trajectories.

Although higher rainfall showed a modest and seemingly counterintuitive association with increased odds of adverse child health outcomes, this likely reflects the dual and context-dependent nature of precipitation in Nigeria. Whilst moderate rainfall supports agricultural productivity and food security (44), excessive rainfall, particularly in the flood-prone southern coastal states, which recorded the highest annual rainfall, contaminates water sources, disrupts sanitation infrastructure, and increases exposure to waterborne diseases (45). Tiwari et al. showed that an overall positive association between rainfall and child weight in Nepal masks simultaneous offsetting processes, with above-average rainfall associated with both increased disease transmission and improved crop production, effects that can act in opposing directions on nutritional status (46). The wealthier southern states also exhibit different sociodemographic profiles from the arid north, making direct climatic comparisons complex. The significant negative interaction between temperature and rainfall for the underweight outcome suggests that the deleterious effect of high temperatures on child nutritional status may be partially attenuated in wetter environments, potentially through better agricultural output and reduced heat stress (47). This interaction warrants further investigation, as it implies that climate adaptation strategies targeting water availability and food systems could help moderate the worst nutritional impacts of heat exposure.

The finding that higher daytime temperatures were associated with greater postpartum distress scores is noteworthy and adds to the limited literature on climate change and maternal mental health in LMICs (48). Several mechanisms may underlie this association. Heat exposure is known to disrupt sleep quality, which is already compromised in the postpartum period, and poor sleep is a well-established risk factor for postpartum depression and anxiety (49). Okun et al. found that poor sleep quality at 6 months postpartum was significantly associated with greater symptoms of both depression (*β* = 0.496, *p* < 0.001) and anxiety (*β* = 0.530, *p* < 0.001) on standardised measures (50), supporting sleep quality as a risk factor for negative maternal affect in the postpartum period. Thermal stress also activates the hypothalamic–pituitary–adrenal axis, elevating cortisol levels and potentially exacerbating mood dysregulation in vulnerable postpartum women (51, 52). Beyond direct physiological pathways, the economic and subsistence pressures that accompany drought and heat stress, including crop failure, food insecurity, and displacement, represent potent psychosocial stressors that compound the psychological burden of early motherhood (51).

The negative associations between both higher temperatures and higher rainfall with the healthcare access composite index represent one of the important findings of this study to improve the healthcare system affected by climate change. A review of climate change and health in Africa notes that climate-related events can directly impact health system supply and delivery, including storm damage to clinic or hospital infrastructure and road damage preventing healthcare staff from reaching their workplaces, whilst also finding that extreme heat and drought disproportionately affect Ethiopia and Nigeria (53). This likely reflects the combined burden of physical distance to facilities, heat-induced fatigue that compounds travel challenges (54), and the prioritisation of immediate subsistence needs, such as water and food security, over preventive healthcare in environmentally stressed households (2). The negative association between higher rainfall and healthcare access, whilst smaller in magnitude, may reflect the role of flooding in disrupting road networks and facility operations, reducing physical access to care at critical moments in the antenatal and peripartum period (45). Notably, a significant positive interaction between temperature and rainfall was detected for healthcare access, suggesting that in wetter environments, the detrimental effect of heat on care-seeking may be partially offset, potentially because moisture availability supports agricultural livelihoods and reduces the acute subsistence pressures that compete with healthcare utilisation.

The associations between climate exposures and maternal anaemia were more modest than those observed for child health outcomes, though not entirely absent. The direction of the rainfall effect may again reflect the dual nature of precipitation in Nigeria: in the high-rainfall southern coastal states, flooding and exposure to waterborne pathogens can compound existing nutritional deficiencies and disrupt the supply of iron-rich foods, potentially elevating anaemia risk through environmental and food system pathways (45). The high prevalence of anaemia in this sample reflects the well-documented burden of iron deficiency, malaria, and nutritional deficiencies among Nigerian women of reproductive age (33). The pathway from climate to anaemia is likely more indirect than for child nutritional outcomes, mediated through nutritional status, malaria transmission, and healthcare access rather than through a single direct physiological mechanism. Future studies incorporating data on malaria status, dietary intake, and iron supplementation could more clearly delineate these pathways. The protective association of secondary and higher maternal education with anaemia is consistent with established literature linking educational attainment to improved nutritional knowledge, dietary diversity, and health-seeking behaviour (34).

Across all models, household wealth and maternal education emerged as strong and consistent modifiers, underscoring the extent to which socioeconomic vulnerability amplifies susceptibility to climate-related health risks (12). This protective effect reflects the role of economic resources in buffering climate shocks through access to improved water and sanitation, dietary diversity, healthcare utilisation, and physical housing quality that reduces heat stress (55). The intersection of poverty and climate exposure represents a critical leverage point for policy intervention: households that are already economically marginalised and residing in the hotter, drier northern states face a compounding disadvantage that cannot be addressed through climate adaptation strategies alone, but requires integrated social protection responses. The divergent patterns observed for rural versus urban residence, specifically the higher odds of stunting but lower odds of wasting in rural areas, likely reflect differences in the chronicity versus acuity of nutritional deficits between settings, and may also be confounded by differences in access to healthcare for diagnosis and treatment (56).

### Strengths and limitations

This study has several important strengths. The use of nationally representative DHS data, combined with geospatially linked climate covariates, enables population-level inference across a large, climatically diverse country. The multilevel analytical framework appropriately accounts for the hierarchical clustering of individuals within communities. The study also examines a broader set of outcomes than most prior work in this area, spanning child nutrition, infectious disease, maternal mental health, anaemia, and healthcare access within a single analytical framework, enabling comparison of climate-health pathways across multiple domains simultaneously.

Nevertheless, several limitations warrant careful consideration. First, the cross-sectional design of the DHS prevents causal inference; the observed associations between climate exposures and health outcomes could be confounded by unmeasured variables that are both spatially correlated with climate and independently associated with health. These unmeasured factors include local sanitation infrastructure, food system characteristics, historical patterns of healthcare investment, and crucial socio-cultural dynamics, such as social support, community resilience, and gender norms, which uniquely shape maternal outcomes. Second, the four-year temporal gap between the climate exposure measurements (2020) and the health outcome assessments (2024). We explicitly acknowledge that environmental conditions in 2020 may not perfectly reflect the exact exposures experienced from 2021 to 2024, particularly for younger children who lacked gestational exposure to that specific period. Furthermore, 2020 was a globally atypical year marked by socio-economic disruptions from the COVID-19 pandemic and unique meteorological anomalies across parts of sub-Saharan Africa, which may limit the representativeness of that baseline. Crucially, because our climate exposure variables are derived from annual averages within the geospatial dataset, our models are unable to account for critical developmental windows, such as trimester-specific exposure during gestation, or short-term extreme events like flash floods and acute heatwaves. Because more recent validated geospatial covariate files within the DHS framework were unavailable, a sensitivity analysis using temporally closer climate data could not be performed. This temporal gap and the reliance on annual aggregations introduce the potential for ecological exposure misclassification. However, because this measurement error is independent of individual-level health status, the misclassification is strictly non-differential. In multi-level mixed-effects regression models, non-differential misclassification typically biases effect estimates towards the null, indicating that our reported associations are likely conservative and may underestimate the true magnitude of the climate-health relationships. Third, the high rates of missing data for several outcomes (notably stunting, wasting, and the maternal health modules, with over 60% missing for some nutritional indicators) raise important concerns regarding a potential reduction in statistical power and the introduction of selection bias. This substantial level of missingness warrants careful consideration regarding the overall robustness of our reported findings. Nevertheless, the fact that our multi-level models retain strong statistical significance across multiple domains despite this reduced power demonstrates the fundamental robustness and stability of the underlying climate-health relationships identified within the population. Fourth, as noted, the postpartum distress measure has not been formally validated against clinical diagnoses of postpartum depression in the Nigerian context, and its use as a proxy for this outcome should be treated with appropriate caution. Fifth, a notable limitation involves our exposure metric for ambient heat, which relied on satellite-derived daytime LST. Although LST provides comprehensive, high-resolution spatial coverage across all clusters, it represents surface skin temperature rather than ambient air temperature, with discrepancies reaching up to 10 to 15 °C depending on local canopy cover and environmental characteristics. Crucially, LST correlates poorly with indoor temperatures, which constitute the primary exposure environment for neonates, young infants, and recently delivered mothers vulnerable to postpartum distress. This structural difference introduces a degree of exposure misclassification into our models. However, because this measurement error is independent of individual-level clinical outcomes, it functions as a non-differential misclassification. This type of error typically attenuates the estimated coefficients, driving our odds ratios towards the null and suggesting that the true adverse impacts of thermal stress on maternal and infant health may be more pronounced than our reported models indicate. Finally, a further limitation concerns the classification of maternal anaemia. The DHS Programme applies a uniform haemoglobin threshold of 12.0 g/dL to all non-pregnant individuals, including those in the immediate postpartum phase. This diverges from WHO public health surveillance guidelines, which recommend a lower threshold of <11.0 g/dL for women in the first 6 weeks after delivery, reflecting their physiological similarity to pregnant women (31). Because the DHS biomarker repository does not feature a dedicated variable to isolate this brief postpartum window, applying a distinct WHO-recommended threshold to this subgroup was unfeasible. Consequently, women in the immediate postpartum period with haemoglobin values between 11.0 and 12.0 g/dL were classified as anaemic under the DHS threshold, even though they would not have met the WHO postpartum criterion, potentially leading to modest overdiagnosis of anaemia in this subgroup. Nevertheless, this classification error operates as a non-differential misclassification with respect to environmental exposures. In macro-level multi-level modelling, non-differential misclassification typically biases the estimated coefficients towards the null, implying that our reported odds ratios serve as conservative estimates and the true impacts of climate stress on maternal anaemia might be stronger than estimated.

### Policy implications

These findings carry significant implications for health policy in Nigeria and for broader efforts to integrate climate adaptation into maternal and child health programmes across the African region. The consistent concentration of climate-related health risk among poorer, less-educated, and rural households points to the need for climate-sensitive social protection programmes that target the most vulnerable communities. In northern Nigeria, particularly, where heat stress is most severe and socioeconomic disadvantage most entrenched, investments in nutritional supplementation, community-based management of acute malnutrition, and heat-resilient housing may deliver co-benefits for child health outcomes. The finding that climate exposures are associated with reduced maternal healthcare access has direct implications for the design of antenatal and postnatal care delivery: mobile outreach services, community health worker programmes, and financial incentives for facility-based care may help maintain utilisation during periods of climate-related disruption. Addressing maternal mental health as a distinct component of postnatal care in climate-vulnerable settings, rather than as an ancillary concern, is also warranted given the evidence presented here. Current climate-related policy frameworks include the National Adaptation Strategy and Plan of Action on Climate Change for Nigeria (NASPA-CCN), the National Climate Change Policy for Nigeria 2021–2030, the Climate Change Act 2021, Nigeria’s updated Nationally Determined Contributions (NDCs), the National Adaptation Plan process, and the Climate Change and Health National Adaptation Plan (HNAP) 2025–2030 (57, 58). The National Climate Change Policy 2021–2030 provides an overarching framework for a low-carbon, climate-resilient, and gender-responsive development pathway. It explicitly identifies health as a climate-sensitive sector and calls for climate-resilient and quality health facilities, including climate-smart healthcare, renewable energy for health infrastructure, improved sanitation and water supply, and stronger health-system resilience to epidemic and pandemic risks (59). Nigeria’s Climate Change and Health National Adaptation Plan (HNAP) 2025–2030, with a focus on disease management, infrastructure resilience, early warning systems and capacity building, is the country’s premier policy blueprint for building climate-resilient health systems. The framework outlines evidence-based interventions to protect citizens from climate-induced diseases, rising temperatures, and extreme weather events. The Climate Change Act 2021 further strengthens this architecture by establishing the National Council on Climate Change as the apex body for climate governance and by mandating economy-wide climate action across sectors. The updated NDCs and the emerging NAP process provide additional channels through which climate adaptation priorities can be translated into sectoral planning, financing, and implementation.

However, the extent to which maternal and child health has been mainstreamed across these frameworks remains limited. The National Climate Change Policy recognises women, children, youth, older persons, persons with disabilities, smallholder farmers, poor households, and pregnant women as vulnerable groups, and it calls for gender mainstreaming, reproductive health, family planning, and the inclusion of vulnerable populations in climate planning. Yet these commitments remain framed largely at the level of broad vulnerability and social inclusion rather than as a detailed maternal and child health adaptation agenda. Similarly, the NDCs and HNAP acknowledge health-sector vulnerability, but maternal nutrition, childhood undernutrition, antenatal and postnatal care disruption, child febrile illness, and postpartum mental health are not yet treated as core climate-sensitive outcomes requiring dedicated indicators, financing, or delivery strategies.

The quantified associations presented in this study, linking ambient temperature variations to stunting, wasting, childhood fever, postpartum distress, and restricted healthcare access, provide a robust empirical baseline that was previously absent from Nigeria’s climate-health evidence base. These findings therefore offer a concrete entry point for the next review cycle of the HNAP, NDCs, and National Climate Change Policy, as well as for the development of future climate-health implementation frameworks. Specifically, they support the integration of gender-responsive and child-centred adaptation strategies into climate budgetary allocations, primary healthcare delivery, nutrition programming, maternal mental health services, routine health information systems, and community-based climate resilience planning. Doing so would move maternal and child health from implicit recognition within vulnerability language to explicit inclusion within Nigeria’s climate adaptation priorities.

## Conclusion

Using nationally representative data from the 2024 Nigeria DHS linked to geospatial climate covariates, this study provides evidence that higher ambient temperatures are associated with worse nutritional outcomes and greater odds of fever in young children, increased maternal postpartum distress, and reduced access to maternal healthcare. These associations are substantially amplified by poverty and low maternal education, highlighting the inequitable distribution of climate-related health burden in Nigeria. As global temperatures continue to rise and climate variability intensifies, the health toll on pregnant women and young children in high-exposure, low-resource settings such as northern Nigeria is likely to escalate unless targeted interventions are implemented. Future longitudinal and experimental research is needed to clarify the causal mechanisms underlying these associations and to evaluate the effectiveness of climate-sensitive health interventions in this context.

## Data Availability

Publicly available datasets were analysed in this study. This data can be found at: Repository Name: The Demographic and Health Surveys (DHS) Programme. Direct Link to Data: https://dhsprogram.com/data/available-datasets.cfm. Accession Numbers: Not applicable; the dataset is identified as the 2024 Nigeria Demographic and Health Survey (DHS). Please note that whilst the data is publicly available, users are typically required to register and submit a research project description to The DHS Programme to gain official access to the datasets.
