# Supplementary material for: Ambient temperature, rainfall, and adverse maternal and child health outcomes in Nigeria: evidence from a national cross-sectional study
Source: Front Public Health. 2026 Jun 29;14:1841642. doi: 10.3389/fpubh.2026.1841642 (PMC13357404; doi:10.3389/fpubh.2026.1841642)
Supplement: Supplementary file 1 [file Table_1.DOCX]

**Supplementary Table S1. Bivariate associations between background characteristics and child health outcomes**

| Variable | Stunting |  | Wasting |  | Underweight |  | Fever |  |
| --- | --- | --- | --- | --- | --- | --- | --- | --- |
|  | **% Stunted** | **p-value** | **% Wasted** | **p-value** | **% Underweight** | **p-value** | **% with Fever** | **p-value** |
| Household wealth |  | <0.001 |  | 0.086 |  | <0.001 |  | 0.004 |
| Poorest | 55.6 |  | 9.5 |  | 38.6 |  | 14.2 |  |
| Poorer | 51.7 |  | 7.6 |  | 32.1 |  | 16.5 |  |
| Middle | 40.5 |  | 6.9 |  | 25.1 |  | 18 |  |
| Richer | 30.2 |  | 9.4 |  | 22 |  | 16.4 |  |
| Richest | 14.8 |  | 8.7 |  | 12.2 |  | 14.5 |  |
| Mother’s education |  | <0.001 |  | 0.101 |  | <0.001 |  | 0.01 |
| No education | 55.1 |  | 8.8 |  | 36.1 |  | 15 |  |
| Primary | 40.5 |  | 7.7 |  | 26.1 |  | 17.9 |  |
| Secondary | 28.8 |  | 9 |  | 20.8 |  | 17 |  |
| Higher | 14 |  | 6.3 |  | 9.8 |  | 14.7 |  |
| Place of residence |  | <0.001 |  | 0.03 |  | <0.001 |  | 0.538 |
| Urban | 27.8 |  | 9.4 |  | 21.5 |  | 15.6 |  |
| Rural | 47.4 |  | 7.7 |  | 30 |  | 16.1 |  |
| Child sex |  | <0.001 |  | 0.297 |  | 0.136 |  | 0.309 |
| Male | 42.1 |  | 8.8 |  | 27.2 |  | 16.2 |  |
| Female | 36.1 |  | 8.1 |  | 25.6 |  | 15.6 |  |
| Continuous variables | **Mean (SD)†** |  | **Mean (SD)†** |  | **Mean (SD)†** |  | **Mean (SD)†** |  |
| Daytime Temperature | 33.6 (2.4) | <0.001 | 33.1 (2.6) | 0.419 | 33.5 (2.5) | <0.001 | 33.1 (2.5) | 0.004 |
| Annual Rainfall | 1154 (429) | <0.001 | 1283 (516) | 0.565 | 1178 (462) | <0.001 | 1235 (506) | 0.005 |
| Child age in months | 31.7 (16.2) | <0.001 | 22.3 (16.2) | <0.001 | 29.8 (17.1) | 0.008 | 27.8 (16.6) | <0.001 |
| Maternal Parity (Total Children) | 4.4 (2.6) | <0.001 | 3.9 (2.5) | 0.113 | 4.3 (2.5) | <0.001 | 4.1 (2.5) | 0.703 |

* Note: For categorical variables, values represent the weighted prevalence (%) of the condition within the group. For continuous predictors, values represent the Mean ± SD of children with the condition. † Standard Deviation (SD) is unweighted.

**Supplementary Table S2. Bivariate associations between background characteristics and maternal health outcomes**

| Variable | Anaemia |  | Postpartum Distress |  | Healthcare Access |  |
| --- | --- | --- | --- | --- | --- | --- |
|  | **% Anaemia** | **p-value** | **Mean (SD)** | **p-value** | **Mean (SD)** | **p-value** |
| Household wealth |  | 0.001 |  | 0.066 |  | <0.001 |
| Poorest | 49.1 |  | -0.04 |  | -0.89 |  |
| Poorer | 52.2 |  | -0.07 |  | -0.57 |  |
| Middle | 46.9 |  | -0.01 |  | -0.01 |  |
| Richer | 47.2 |  | 0.01 |  | 0.67 |  |
| Richest | 40.5 |  | 0.01 |  | 1.23 |  |
| Mother’s education |  | <0.001 |  | 0.001 |  | <0.001 |
| No education | 50.1 |  | -0.07 |  | -0.82 |  |
| Primary | 51.1 |  | -0.01 |  | -0.05 |  |
| Secondary | 45.9 |  | 0.01 |  | 0.68 |  |
| Higher | 38.1 |  | 0.07 |  | 1.25 |  |
| Place of residence |  | <0.001 |  | 0.414 |  | <0.001 |
| Urban | 43.9 |  | -0.01 |  | 0.64 |  |
| Rural | 49.9 |  | -0.03 |  | -0.48 |  |
| Continuous variables | **Mean (SD)†** |  | **β** |  | **β** |  |
| Daytime Temperature | 33.1 (2.6) | 0.895 | 0.008 | 0.095 | -0.14 | <0.001 |
| Annual Rainfall | 1256 (486) | 0.695 | -0.00002 | 0.393 | 0.001 | <0.001 |
| Mother’s age in years | 30.1 (6.7) | 0.636 | -0.005 | 0.001 | 0.015 | <0.001 |
| Total children | 4.2 (2.5) | 0.013 | -0.014 | <0.001 | -0.089 | <0.001 |
| Child age in months | 29.5 (17.9) | 0.08 | -0.003 | <0.001 | 0.001 | 0.367 |

* Note: For categorical variables, the Anaemia column reports weighted prevalence (%), while the Distress and Healthcare columns report Mean scores. For continuous predictors (Mother's age, Total children, Child age): the Anaemia column reports the Mean ± SD of anaemic mothers; the Distress and Healthcare columns report the linear regression coefficient (β). † Standard Deviation (SD) is unweighted.
